# Factors Associated with Low-Level Viraemia and Virological Failure: Results from the Austrian HIV Cohort Study

**DOI:** 10.1371/journal.pone.0142923

**Published:** 2015-11-13

**Authors:** Gisela Leierer, Katharina Grabmeier-Pfistershammer, Andrea Steuer, Maria Geit, Mario Sarcletti, Bernhard Haas, Manfred Kanatschnig, Michaela Rappold, Robert Zangerle, Bruno Ledergerber, Ninon Taylor, Austrian HIV Cohort Study Group

**Affiliations:** 1 Department of Dermatology and Venereology, Medical University of Innsbruck, Innsbruck, Austria; 2 Austrian HIV Cohort Study, Innsbruck, Austria; 3 Department of Dermatology, Medical University of Vienna, Vienna, Austria; 4 Otto-Wagner Hospital, Department of Pulmonology, Vienna, Austria; 5 Department of Dermatology, General Hospital Linz, Linz, Austria; 6 Department of Internal Medicine, General Hospital Graz-West, Graz, Austria; 7 1^st^ Medical Department, General Hospital Klagenfurt, Klagenfurt, Austria; 8 Division of Infectious Diseases and Hospital Epidemiology, University Hospital Zurich, University of Zurich, Zurich, Switzerland; 9 Department of Internal Medicine III with Hematology, Medical Oncology, Hemostaseology, Infectious Diseases, Rheumatology, Oncologic Center, Laboratory of Immunological and Molecular Cancer Research, Paracelsus Medical University, Salzburg, Austria; University of British Columbia, CANADA

## Abstract

**Background:**

In human immunodeficiency virus treatment adequate virological suppression is warranted, nevertheless for some patients it remains a challenge. We investigated factors associated with low-level viraemia (LLV) and virological failure (VF) under combined antiretroviral therapy (cART).

**Materials and Methods:**

We analysed patients receiving standard regimens between 1^st^ July 2012 and 1^st^ July 2013 with at least one viral load (VL) measurement below the quantification limit (BLQ) in their treatment history. After a minimum of 6 months of unmodified cART, the next single VL measurement within 6 months was analysed. VF was defined as HIV RNA levels ≥200 copies/mL and all other quantifiable measurements were classified as LLV. Factors associated with LLV and VF compared to BLQ were identified by logistic regression models.

**Results:**

Of 2276 participants, 1972 (86.6%) were BLQ, 222 (9.8%) showed LLV and 82 (3.6%) had VF. A higher risk for LLV and VF was shown in patients with cART interruptions and in patients with boosted PI therapy. The risk for LLV and VF was lower in patients from centres using the Abbott compared to the Roche assay to measure VL. A higher risk for LLV but not for VF was found in patients with a higher VL before cART [for >99.999 copies/mL: aOR (95% CI): 4.19 (2.07–8.49); for 10.000–99.999 copies/mL: aOR (95% CI): 2.52 (1.23–5.19)] and shorter cART duration [for <9 months: aOR (95% CI): 2.59 (1.38–4.86)]. A higher risk for VF but not for LLV was found in younger patients [for <30 years: aOR (95% CI): 2.76 (1.03–7.35); for 30–50 years: aOR (95% CI): 2.70 (1.26–5.79)], people originating from high prevalence countries [aOR (95% CI): 2.20 (1.09–4.42)] and in male injecting drug users [aOR (95% CI): 2.72 (1.38–5.34)].

**Conclusions:**

For both VF and LLV, factors associated with adherence play a prominent role. Furthermore, performance characteristics of the diagnostic assay used for VL quantification should also be taken into consideration.

## Introduction

The advent of combined antiretroviral therapy (cART) resulted in a significant reduction in morbidity and mortality for persons infected with the human immunodeficiency virus type 1 (HIV-1) [1,2]. Suppression of plasma HIV-1 RNA through antiretroviral therapy is the goal of HIV treatment. Adequate virological suppression also hinders the development of resistance mutations. Current guidelines define virological suppression below the limit of quantification of commercial assays which is usually between <20 to 75 copies/mL [3]. On the other hand, the threshold for virological failure (VF) remains controversial ranging from 50 to 200 copies/mL [3,4]. These discrepancies reflect the fact that optimal virological suppression remains often an ideal goal but some patients may show persistent low-level viraemia (LLV) and the best clinical practice in such cases, especially concerning LLV between 20–200 copies/mL, is not resolved. Further, the threshold where detectable viral load (VL) in plasma comes from ongoing replication is not known and remains indistinguishable from circulating virus due to the release of HIV from latently infected cells.

The objective of this study was to investigate factors associated with LLV defined with a cutoff <200 copies/mL and VF in a cohort of HIV-1 positive patients.

## Methods

### Study Design and Data Collection

The Austrian HIV Cohort Study (AHIVCOS) is an open, multicentre, prospective, observational cohort study of HIV-infected individuals followed at seven HIV treatment centres in Austria. The study was initiated in 2001 and patients are enrolled actively and prospectively covering approximately 80% of all treated HIV-infected patients in Austria. Data collection includes sociodemographic information, complete history of cART as well as laboratory results with VL and CD4 cell count measurements, usually repeated every 3–6 months. The laboratory analyses were performed in each single centre and for HIV-1 VL assessment, five centres used the Roche Cobas AmpliPrep/Cobas TaqMan 2.0 assay (Taqman, Roche Diagnostics, Mannheim, Germany), referred to as the Roche assay in this manuscript, while the remaining two centres used the Abbott RealTime HIV-1 assay (Abbott RT, Abbott Diagnostics, Wiesbaden, Germany), referred to as the Abbott assay, during the period of time analysed.

Approval for this study was obtained from the local ethical committees of all participating centres: ethics committee of the Vienna Medical University (No. 898/ 2010), of the Salzburg Federal Government (No. 1159/ 2010), of the Graz Medical University (No. 21-431/ 2010), of the Innsbruck Medical University (No 283/4.4/ 2009), of the Upper Austria Federal Government (No C-3-10/ 2010) and of the Carynthian Federal State (No A-13-11/2011). Written informed consent was given by the patients for their information to be stored in the hospital database and used for research.

### Statistical Analysis

#### Patient selection

A retrospective analysis was conducted on individuals receiving unmodified cART for more than 6 months between 1^st^ July 2012 and 1^st^ July 2013 enrolled in AHIVCOS (N = 4289). Patients who were not on stable cART due to either interruptions or switches were excluded (N = 290). During these 6 months, patients had to receive cART with 2 nucleoside reverse-transcriptase inhibitors (NRTIs) and either a non-nucleoside reverse-transcriptase inhibitor (NNRTI) or a boosted protease inhibitor (PI) or an integrase inhibitor (INSTI). Individuals with other cART regimens than those mentioned above, were excluded (N = 489). A further exclusion criterion was a lacking VL below the respective limit of quantification in their treatment history. Thus, another 461 individuals were excluded from analyses (35 tested with the Abbott assay and 426 with the Taqman assay).

In total, 3049 patients fulfilled the inclusion criteria. 773 patients did not have any VL measurement between 6 and 12 months after initiation of the respective cART regimen and were too excluded. 2276 patients remained and their first VL measurement between 6 and 12 months after initiation of the respective cART regimen was evaluated.

#### Definition of LLV and VF

All quantifiable HIV VL measurements below <200 copies/mL were classified as LLV. For further analyses, LLV was divided into two categories, namely HIV RNA levels of 51–199 copies/mL and HIV RNA levels of ≤50 copies/mL. VF was defined as HIV RNA levels ≥ 200 copies/mL.

#### Statistical methods

Groups were compared using χ² test or Fisher´s exact test where appropriate. To investigate the association between various demographic and clinical parameters with LLV as well as VF compared to BLQ, two separate univariable and multivariable logistic regression models were performed. As reference category for both analyses, patients who were BLQ at baseline were used (N = 1972). For the first analysis, to find factors associated with LLV, 82 patients with VF were excluded. 222 (10.1%) of the remaining 2194 patients had LLV below 200 copies/mL. For the second analysis, to find factors associated with VF, 222 patients who had LLV below 200 copies/mL were excluded. 82 (4.0%) of the remaining 2054 patients had a VF. Variables considered for univariable analyses were age at VL measurement, HIV transmission category, nationality, CD4 count before cART, ever cART interruptions, type of VL assay used, diabetes, cART regimen, VL before cART and cART duration. Multivariable models were adjusted for demographic variables age at VL measurement and HIV transmission category whether or not significant in univariable analyses. Further adjustment was made for variables with p <0.05 in univariable analyses. Ever cART interruptions were defined as interruptions prior to 6 months of stable cART of the respective cART regimen. A cART discontinuation for at least 8 days after having started cART was regarded as cART interruption.

In addition, different sensitivity analyses were performed for different criteria in patient selection. Another sensitivity analysis was conducted in order to analyse patients´ characteristics stratified by cART regimen. Further, we additionally did an analysis for the two outcomes LLV and VF excluding the centres measuring VL with the Abbott assay. P-values of <0.05 were considered to indicate statistical significance.

All analyses were conducted using Stata software, version 13.1 (StataCorp, College Station, TX, USA).

## Results

### Patient characteristics stratified by VL

**Fig 1:**
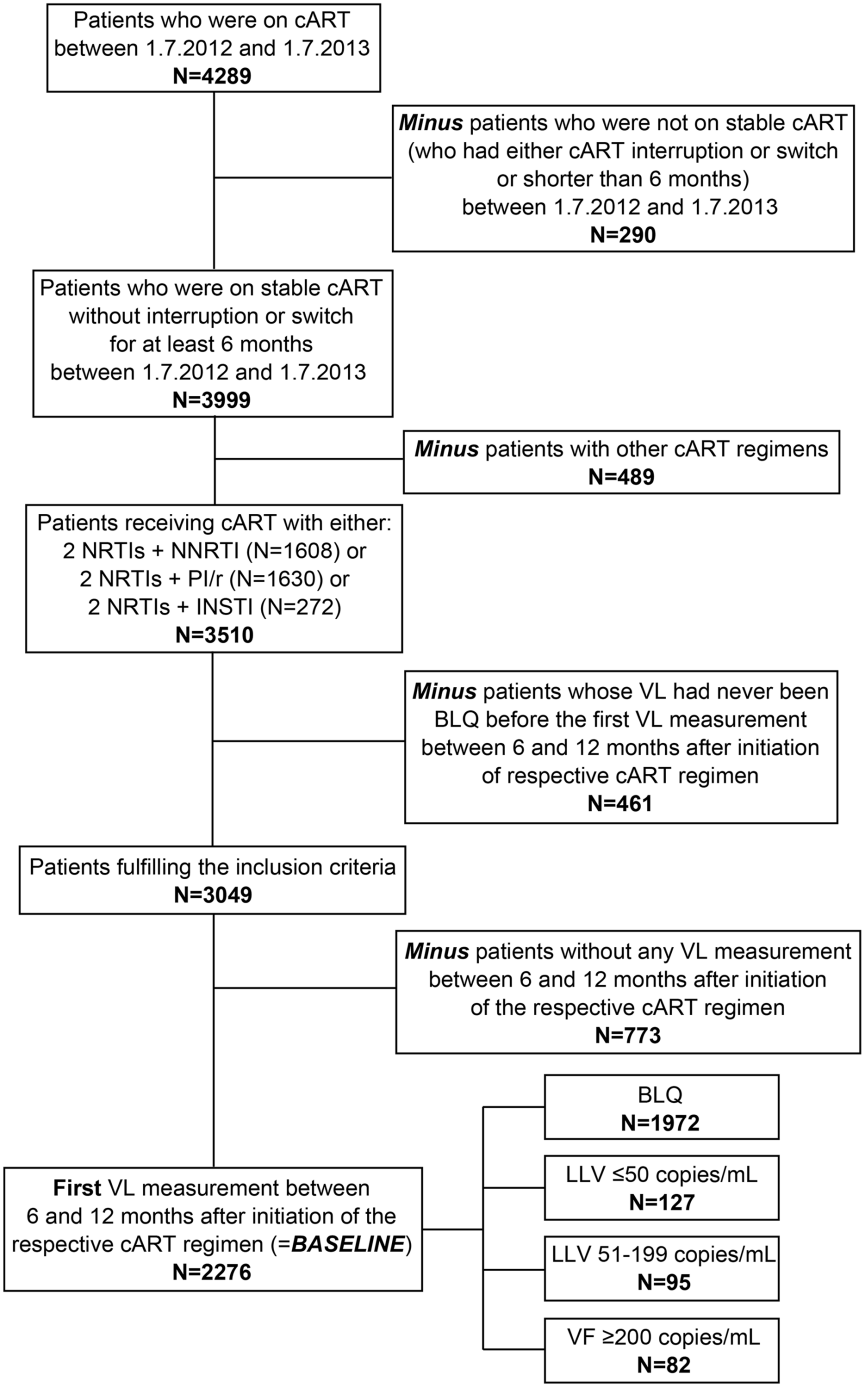
Flowchart of patient selection.

The flowchart of the patient selection is shown in Fig 1 and the characteristics of the 2276 included participants, stratified by HIV RNA levels are listed in Table 1. For all the patients included in the study, the median age was 43.8 years (interquartile range IQR: 36.6–51.6) and the majority were men (72.1%). cART was PI-based in 42.9% (976/2276) of the patients, with darunavir being mostly used. NNRTI- and INSTI-based therapies were used in 57.1% (1300/2276) of the cases. The median time on cART was 68.4 months (IQR: 32.0–129.8). Seventy eight percent of the participants had their HIV RNA measured by the Roche assay and 22% had their HIV RNA measured using the Abbott assay. 773 did not have a VL measurement within 12 months of respective cART initiation and were excluded. 377 (16.6%) patients had no prior cART. 1972 of the 2276 included patients were BLQ (86.4%), while 222 (9.8%) showed a LLV and 82 (3.6%) had VF. Stratification by VL of sociodemographic and laboratory analyses showed a higher frequency of male injecting drug users (12.6 and 22.0 versus 8.8%) and of patients with cART interruptions (32.0 and 48.8 versus 24.1%) in the LLV and VF group compared to patients who were BLQ. PI-based regimens were more frequent (65.9 and 51.4 versus 41.0%). Patients whose HIV RNA was measured by the Roche assay rather than by the Abbott assay had more quantifiable VL measurements ≥200 copies/mL and below 200 copies/mL compared to individuals who were BLQ (96.3 and 90.5 versus 75.9%). A higher VL before cART (>99.999 copies/mL: 50.0% versus 36.9%) was more likely to be found in patients with LLV. Patients with VF were younger (<30 years: 12.2 versus 8.5%; 30–50 years: 78.1 versus 64.8%) and more likely originating from high prevalence countries (18.3 versus 8.8%). Concerning VL cases, the majority (35.4%) of the VF cases were below 1000 copies/mL and only 6.1% reached very high VLs (>99.000 copies/mL).

**Table 1:** Characteristics of patients stratified by HIV RNA levels below the limit of quantification and low-level viraemia defined as quantifiable HIV RNA levels <200 copies/mL and virological failure defined as HIV RNA levels ≥200 copies/mL.

	All patients	BLQ	LLV <200	VF ≥200		
No. of patients	N = 2276	N = 1972	N = 222	N = 82		
	N (%)	N (%)	N (%)	N (%)	P value^1^	P value^2^
**Age at VL measurement**					0.648	0.002
<30 years	201 (8.8)	168 (8.5)	23 (10.4)	10 (12.2)		
30–50 years	1481 (65.1)	1277 (64.8)	140 (63.1)	64 (78.1)		
>50 years	594 (26.1)	527 (26.7)	59 (26.6)	8 (9.8)		
**HIV transmission category**					0.034	0.001
Male injecting drug user	220 (9.7)	174 (8.8)	28 (12.6)	18 (22.0)		
Female injecting drug user	95 (4.2)	85 (4.3)	5 (2.3)	5 (6.1)		
Male heterosexual	477 (21.0)	421 (21.4)	45 (20.3)	11 (13.4)		
Female heterosexual	507 (22.3)	448 (22.7)	35 (15.8)	24 (29.3)		
Other	98 (4.3)	82 (4.2)	13 (5.9)	3 (3.7)		
Men who have sex with men	879 (38.6)	762 (38.6)	96 (43.2)	21 (25.6)		
**Nationality**					0.448	0.004
High prevalence country	212 (9.3)	174 (8.8)	23 (10.4)	15 (18.3)		
Low prevalence country	2064 (90.7)	1798 (91.2)	199 (89.6)	67 (81.7)		
**CD4 count before cART**					0.349	0.770
Missing	346 (15.2)	295 (15.0)	39 (17.6)	12 (14.6)		
<50 cells/μL	233 (10.2)	207 (10.5)	21 (9.5)	5 (6.1)		
50–199 cells/μL	465 (20.4)	399 (20.2)	48 (21.6)	18 (22.0)		
200–349 cells/μL	716 (31.5)	615 (31.2)	75 (33.8)	26 (31.7)		
≥350 cells/μL	516 (22.7)	456 (23.1)	39 (17.6)	21 (25.6)		
**Ever cART interruptions** ^3^					0.010	<0.001
≥1	587 (25.8)	476 (24.1)	71 (32.0)	40 (48.8)		
None	1689 (74.2)	1496 (75.9)	151 (68.0)	42 (51.2)		
**Assay used**					<0.001	<0.001
Abbott RealTime (2 centres)	501 (22.0)	475 (24.1)	23 (10.4)	3 (3.7)		
Roche TaqMan 2.0 (5 centres)	1775 (78.0)	1497 (75.9)	199 (90.5)	79 (96.3)		
**Ever diabetes** ^4^					0.246	0.767
Yes	89 (3.9)	75 (3.8)	12 (5.4)	2 (2.4)		
No	2187 (96.1)	1897 (96.2)	210 (94.6)	80 (97.6)		
**cART regimen**					0.003	<0.001
2 NRTIs + PI/r	976 (42.9)	808 (41.0)	114 (51.4)	54 (65.9)		
2 NRTIs + NNRTI/INSTI	1300 (57.1)	1164 (59.0)	108 (48.7)	28 (34.2)		
**VL before cART**					<0.001	0.746
Missing	406 (17.8)	353 (17.9)	39 (17.6)	14 (17.1)		
>99.999 copies/mL	867 (38.1)	728 (36.9)	111 (50.0)	28 (34.2)		
10.000–99.999 copies/mL	753 (33.1)	658 (33.4)	63 (28.4)	32 (39.0)		
≤9.999 copies/mL	250 (11.0)	233 (11.8)	9 (4.1)	8 (9.8)		
**cART duration** ^5^					0.056	0.781
<9 months	86 (3.8)	69 (3.5)	15 (6.8)	2 (2.4)		
9–18 months	183 (8.0)	158 (8.0)	17 (7.7)	8 (9.8)		
>18 months	2007 (88.2)	1745 (88.5)	190 (85.6)	72 (87.8)		
**First-line cART** ^6^					0.949	0.091
Yes	377 (16.6)	332 (16.8)	37 (16.7)	8 (9.8)		
No	1899 (83.4)	1640 (83.2)	185 (83.3)	74 (90.2)		

Abbreviations: LLV, low-level viraemia; VF, virological failure; VL, viral load; NRTIs, nucleoside reverse transcriptase inhibitors; NNRTI, non-nucleoside reverse transcriptase inhibitor; PI/r, boosted protease inhibitor; INSTI, integrase inhibitor; BLQ, below the limit of quantification; cART, combination antiretroviral therapy;

^1^ Comparison between quantifiable HIV RNA levels <200 copies/mL (LLV) and HIV RNA levels below the limit of quantification (BLQ).

^2^ Comparison between HIV RNA levels ≥200 copies/mL (VF) and HIV RNA levels below the limit of quantification (BLQ).

^3^ Interruptions prior to 6 months stable cART of the respective cART regimen.

^4^ Diabetes mellitus prior to 6 months stable cART of the respective cART regimen.

^5^ cART duration until 6 months stable cART of the respective cART regimen.

^6^ Whether the respective cART regimen is a first-line cART or not.

### Correlates of LLV

As shown in Table 2, a reduction in risk for LLV was found in female heterosexuals compared to men who have sex with men [adjusted Odds Ratio, aOR (95% CI): 0.62 (0.41–0.95)] as well as in patients from 2 centres which used the Abbott assay as compared to the other centres measuring VL by the Roche assay [aOR (95% CI): 0.33 (0.21–0.51)]. Interrupted cART [aOR (95% CI): 1.69 (1.22–2.34)] versus uninterrupted cART, 2 NRTIs with a boosted PI as cART regimen [aOR (95% CI): 1.54 (1.15–2.06)] versus 2 NRTIs with a NNRTI or INSTI, a higher VL before cART [for >99.999 copies/mL: aOR (95% CI): 4.19 (2.07–8.49); for 10.000–99.999 copies/mL: aOR (95% CI): 2.52 (1.23–5.19); compared to ≤9.999 copies/mL] and a shorter cART duration [<9 months: aOR (95% CI): 2.59 (1.38–4.86) compared to >18 months] increased the risk of LLV.

**Table 2:** Univariable and multivariable logistic regression results: Association between different factors and low-level viraemia as well as virological failure compared to HIV RNA levels below the limit of quantification.

Outcome	LLV <200				VF ≥200			
No. of patients included	N = 2194				N = 2054			
No. of outcomes	N = 222				N = 82			
	Univariable		Multivariable		Univariable		Multivariable	
	OR	(95% CI)	OR	(95% CI)	OR	(95% CI)	OR	(95% CI)
**Age at VL measurement**								
<30 years	1.22	(0.73–2.04)	1.03	(0.60–1.79)	3.92	(1.52–10.10)	2.76	(1.03–7.35)
30–50 years	0.98	(0.71–1.35)	0.97	(0.69–1.35)	3.30	(1.57–6.93)	2.70	(1.26–5.79)
>50 years	1.00	(Reference)	1.00	(Reference)	1.00	(Reference)	1.00	(Reference)
**HIV transmission category**								
Male injecting drug user	1.28	(0.81–2.01)	1.11	(0.69–1.79)	3.75	(1.96–7.20)	2.72	(1.38–5.34)
Female injecting drug user	0.47	(0.18–1.18)	0.43	(0.17–1.12)	2.13	(0.78–5.81)	1.98	(0.70–5.60)
Male heterosexual	0.85	(0.58–1.23)	0.85	(0.58–1.25)	0.95	(0.45–1.99)	0.79	(0.36–1.72)
Female heterosexual	0.62	(0.41–0.93)	0.62	(0.41–0.95)	1.94	(1.07–3.53)	1.09	(0.55–2.16)
Other	1.26	(0.68–2.34)	1.31	(0.69–2.48)	1.33	(0.39–4.55)	1.30	(0.37–4.56)
Men who have sex with men	1.00	(Reference)	1.00	(Reference)	1.00	(Reference)	1.00	(Reference)
**Nationality**								
High prevalence country	1.19	(0.75–1.89)			2.31	(1.29–4.14)	2.20	(1.09–4.42)
Low prevalence country	1.00	(Reference)			1.00	(Reference)	1.00	(Reference)
**CD4 count before cART**								
Missing	1.55	(0.97–2.47)			0.88	(0.43–1.82)		
<50 cells/μL	1.19	(0.68–2.07)			0.52	(0.20–1.41)		
50–199 cells/μL	1.41	(0.90–2.19)			0.80	(0.51–1.86)		
200–349 cells/μL	1.43	(0.95–2.14)			0.92	(0.51–1.65)		
≥350 cells/μL	1.00	(Reference)			1.00	(Reference)		
**Ever cART interruptions** ^1^								
≥1	1.48	(1.09–2.00)	1.69	(1.22–2.34)	2.99	(1.92–4.67)	2.93	(1.84–4.67)
None	1.00	(Reference)	1.00	(Reference)	1.00	(Reference)	1.00	(Reference)
**Assay used**								
Abbott RealTime (2 centres)	0.36	(0.23–0.57)	0.33	(0.21–0.51)	0.12	(0.04–0.38)	0.09	(0.03–0.30)
Roche TaqMan 2.0 (5 centres)	1.00	(Reference)	1.00	(Reference)	1.00	(Reference)	1.00	(Reference)
**Ever diabetes** ^2^								
Yes	1.45	(0.77–2.70)			0.63	(0.15–2.62)		
No	1.00	(Reference)			1.00	(Reference)		
**cART regimen**								
2 NRTIs + PI/r	1.52	(1.15–2.01)	1.54	(1.15–2.06)	2.78	(1.74–4.42)	2.36	(1.45–3.83)
2 NRTIs + NNRTI/INSTI	1.00	(Reference)	1.00	(Reference)	1.00	(Reference)	1.00	(Reference)
**VL before cART**								
Missing	2.86	(1.36–6.02)	2.70	(1.27–5.76)	1.16	(0.48–2.80)		
>99.999 copies/mL	3.95	(1.97–7.91)	4.19	(2.07–8.49)	1.12	(0.50–2.49)		
10.000–99.999 copies/mL	2.48	(1.21–5.06)	2.52	(1.23–5.19)	1.42	(0.64–3.12)		
≤9.999 copies/mL	1.00	(Reference)	1.00	(Reference)	1.00	(Reference)		
**cART duration** ^3^								
<9 months	2.00	(1.12–3.56)	2.59	(1.38–4.86)	0.70	(0.17–2.92)		
9–18 months	0.99	(0.59–1.67)	1.00	(0.58–1.73)	1.23	(0.58–2.59)		
>18 months	1.00	(Reference)	1.00	(Reference)	1.00	(Reference)		
**First-line cART** ^4^								
Yes	0.99	(0.68–1.43)			0.53	(0.26–1.12)		
No	1.00	(Reference)			1.00	(Reference)		

Abbreviations: LLV, low-level viraemia; VF, virological failure; VL, viral load; CI, confidence interval; OR, odds ratio; NRTIs, nucleoside reverse transcriptase inhibitors; NNRTI, non-nucleoside reverse transcriptase inhibitor; PI/r, boosted protease inhibitor; INSTI, integrase inhibitor; cART, combination antiretroviral therapy;

^1^ Interruptions prior to 6 months stable cART of the respective cART regimen.

^2^ Diabetes mellitus prior to 6 months stable cART of the respective cART regimen.

^3^ cART duration until 6 months stable cART of the respective cART regimen.

^4^ Whether the respective cART regimen is a first-line cART or not.

### Correlates of VF

A reduction in risk of VF was found in patients from 2 centres using the Abbott assay as compared to the other centres measuring VL by the Roche assay [aOR (95% CI): 0.09 (0.03–0.30)]. Younger age [for <30 years: aOR (95% CI): 2.76 (1.03–7.35); for 30–50 years: aOR (95% CI): 2.70 (1.26–5.79); compared to >50 years], male intravenous drug users [aOR (95% CI): 2.72 (1.38–5.34)] versus men who have sex with men, originating from high prevalence countries [aOR (95% CI): 2.20 (1.09–4.42)], interrupted cART [aOR (95% CI): 2.93 (1.84–4.67)] compared to uninterrupted cART and 2 NRTIs with a boosted PI as cART regimen [aOR (95% CI): 2.36 (1.45–3.83)] versus 2 NRTIs with a NNRTI or INSTI increased the risk of VF (Table 2).

### Sensitivity analyses

We performed several sensitivity analyses with different criteria concerning patient selection. One analysis was performed without considering a VL measurement BLQ in treatment history in order to exclude patients with very short cART durations. Another analysis was conducted regarding a shorter recruitment period of 6 months instead of 12 months and one analysis including patients receiving unmodified cART for >9 months instead of >6 months. All additional analyses did, however, not reveal any substantial differences in ORs compared to our primary analysis (data not shown). Stratification of the patients by regimen showed that VL and age at baseline, HIV transmission category, nationality, CD4 cell count before cART, prior cART interruptions, assay used and VL before cART differ significantly (see S1 Table). In addition, sensitivity analyses were conducted for the two outcomes (LLV <200 copies/mL and VF ≥200 copies/mL) excluding the samples analysed with the Abbott assay. The exclusion of patients whose VL had been measured by the Abbott assay did not reveal any major changes in HRs compared to the original models (see S2 Table).

## Discussion

This study of well-defined patients on stable cART over a period of more than 6 months gives insights into the different factors associated with LLV and VF. As expected, adherence-associated factors play a predominant role. Accordingly, cART interruptions constitute an independent risk factor for both LLV and VF, while younger age, the category of male injecting drug users and originating from high prevalence regions are associated with VF alone. Additionally, PI-based regimens appear as independent markers for both groups. Several studies have already shown that NNRTI-based combinations may result in a better suppression of plasma viral load than PI-based cART [5–7]. On the other hand, we have to acknowledge a potential clinician induced bias for favorably prescribing PIs in case of suspected adherence issues. We performed a supplementary stratification of our study patients by regimen and we observed that patients receiving PIs were found significantly more frequently in the group of intravenous drug users and in the group originating from high prevalence regions (see S1 Table). In our study, for first-line therapy, NNRTI-based regimens were the most commonly used (N = 226), followed by PIs (N = 116). INSTIs were less represented due to recent release during the observation period (N = 35). However, the majority of our patients were on 2^nd^ or more lines of therapy and PIs such as NNRTIs were both equally represented (N = 855 and N = 860, respectively), followed again by INSTIs (N = 184).

Further, the association found between high baseline VL and the risk of developing LLV is in line with the hypothesis that the size of the cellular compartment infected before cART initiation determines the level of residual viraemia [6] and is in accordance with other studies [5,8]. Interestingly, the CD4 count before cART start, especially in the very low range of below 50 cells/μl was comparable in all our 3 groups, BLQ, LLV and VF. This observation argues against the concept that LLV may be more frequent in patients presenting at a late stage of infection.

For our group of patients on stable suppressive therapy, we found a significant risk reduction for LLV and VF according to the assay used for VL quantification. This finding is interesting since both the Abbott as well as the Roche assays are widely used in clinical settings to measure HIV RNA and differ significantly regarding their extraction system, primers and probe design. Although the overall correlation between the Roche and Abbott assay in the higher range of quantification is good [9,10], significant discrepancies between these assays have been described at LLV [11–13]. In an international comparative analysis by Swenson et al., the concordance between four commercial assays including the Roche and Abbott assay was determined and it was shown that the inter-assay concordance at a threshold of 200 copies/mL was much higher than at the clinically relevant threshold of 50 copies/mL [14] The published literature is controversial concerning the impact of LLV as a predictor of progressive viral rebound [15–19]. Findings by Ribaudo et al. from a large retrospective analysis found the same predictive value for virological rebound at an HIV RNA of <200 copies/mL as compared to a threshold of <50 copies/mL [18]. These discrepancies may be partly explained due to different definitions of LLV and VF but also due to different quantification assays used. The latter is most problematic since treatment decisions are based on the definitions of VF and virological suppression, all within the lower range of viraemia. Consecutively, if quantification assays are not concordant in such settings, a univocal threshold of VF becomes impossible to establish. Many factors may contribute to such assay discordance, ranging from preanalytic issues, such as handling procedures, to viral blips or different primer target regions [13,20–25]. In our analysis, the Roche assay was predominantly used (78% versus 22% Abbott assay) limiting a comparative analysis. Additionally, due to the multicentre study design, we cannot rule out different local practices as potential further bias.

Finally, concerned by the high number of patients excluded from our study due to missing VL assessment within 6 to 12 months (N = 773), we compared patients´ characteristics of the two groups (data not shown). Individuals excluded differed significantly from included patients in some factors associated with LLV and/or VF. Excluded patients were significantly older, males were more frequently infected through injecting drug use and females through heterosexual contacts. HIV RNA of excluded individuals was more frequently measured by the Roche assay. On the other hand, VL and CD4 count before cART initiation were significantly lower and excluded patients had a shorter overall therapy duration, more cART interruptions and were more frequently on first-line therapy.

In conclusion, the strengths of our study include its large sample size with similar national standards of care and our results support that adherence associated issues play a central part in LLV and VF development. The association between PI-based regimens and risk of LLV and VF remains biased due to confounding by indication. Our data seems to support the concept that performance characteristics of the diagnostic assay used has to be taken into account for evaluation of LLV and especially in cases where clinical decisions such as therapy switches are pending. The present study does not yet include clinical follow-up and predictivity. Despite its observational design, it provides implications for patient management and forms the basis for future outcome studies. We suggest that similar studies with clinical follow-up including different test systems may be able to evaluate the need for guidelines taking into account different performance characteristics of each assay to establish adequate HIV RNA cutoffs.

## Supporting Information

S1 Table Characteristics of patients stratified by regimen. (DOCX)

S2 Table Univariable and multivariable logistic regression results: Association between different factors and low-level viraemia as well as virological failure compared to HIV RNA levels below the limit of quantification. Exclusion of the samples in which the Abbott assay was used.
(DOCX)
