# Supplementary material for: Factors Associated with Low-Level Viraemia and Virological Failure: Results from the Austrian HIV Cohort Study
Source: PLoS One. 2015 Nov 13;10(11):e0142923. doi: 10.1371/journal.pone.0142923 (PMC4643888; doi:10.1371/journal.pone.0142923)
Supplement: S1 Table — (DOCX) [file pone.0142923.s001.docx]

**S1 Table: Characteristics of patients stratified by regimen.**

| Total: N=2276 | **NRTI + NNRTI** | | **NRTI + PI/r** | | **NRTI + INSTI** | |  |
| --- | --- | --- | --- | --- | --- | --- | --- |
| N (%) | **1081** | **(100.0)** | **976** | **(100.0)** | **219** | **(100.0)** | **P value^1^** |
| **Viral load at baseline** |  |  |  |  |  |  | <0.001 |
| VF ≥200 copies/mL | 23 | (2.1) | 54 | (5.5) | 5 | (2.3) |  |
| LLV 51-199 copies/mL | 29 | (2.7) | 54 | (5.5) | 12 | (5.5) |  |
| LLV ≤50 copies/mL | 59 | (5.5) | 60 | (6.2) | 8 | (3.7) |  |
| BLD + BLQ | 970 | (89.7) | 808 | (82.8) | 194 | (88.6) |  |
| **Age at viral load measurement** |  |  |  |  |  |  | <0.001 |
| <30 years | 66 | (6.1) | 113 | (11.6) | 22 | (10.1) |  |
| 30-50 years | 708 | (65.5) | 650 | (66.6) | 123 | (56.2) |  |
| >50 years | 307 | (28.4) | 213 | (21.8) | 74 | (33.8) |  |
| **HIV transmission category** |  |  |  |  |  |  | <0.001 |
| Male injecting drug user | 65 | (6.0) | 133 | (13.6) | 22 | (10.1) |  |
| Female injecting drug user | 27 | (2.5) | 58 | (5.9) | 10 | (4.6) |  |
| Male heterosexual | 236 | (21.8) | 206 | (21.1) | 35 | (16.0) |  |
| Female heterosexual | 227 | (21.0) | 235 | (24.1) | 45 | (20.6) |  |
| Other | 45 | (4.2) | 38 | (3.9) | 15 | (6.9) |  |
| Men who have sex with men | 481 | (44.5) | 976 | (31.4) | 92 | (42.0) |  |
| **Nationality** |  |  |  |  |  |  | 0.010 |
| High prevalence country | 87 | (8.1) | 111 | (11.4) | 14 | (6.4) |  |
| Low prevalence country | 994 | (92.0) | 865 | (88.6) | 205 | (93.6) |  |
| **CD4 count before cART** |  |  |  |  |  |  | 0.035 |
| Missing | 169 | (15.6) | 143 | (14.7) | 34 | (15.5) |  |
| <50 cells/µL | 94 | (8.7) | 106 | (10.9) | 33 | (15.1) |  |
| 50-199 cells/µL | 205 | (19.0) | 213 | (21.8) | 47 | (21.5) |  |
| 200-349 cells/µL | 355 | (32.8) | 308 | (31.6) | 53 | (24.2) |  |
| ≥350 cells/µL | 258 | (23.9) | 206 | (21.1) | 52 | (23.7) |  |
| **Ever cART interruptions^2^** |  |  |  |  |  |  | <0.001 |
| ≥1 | 205 | (19.0) | 310 | (31.8) | 72 | (32.9) |  |
| None | 876 | (81.0) | 666 | (68.2) | 147 | (67.1) |  |
| **Assay used** |  |  |  |  |  |  | <0.001 |
| Abbott RealTime (2 centres) | 190 | (17.6) | 243 | (24.9) | 68 | (31.1) |  |
| Roche TaqMan 2.0 (5 centres) | 891 | (82.4) | 733 | (75.1) | 151 | (69.0) |  |
| **Ever diabetes^3^** |  |  |  |  |  |  | 0.444 |
| Yes | 37 | (3.4) | 41 | (4.2) | 11 | (5.0) |  |
| No | 1044 | (96.6) | 935 | (95.8) | 208 | (95.0) |  |
| **Viral load before cART** |  |  |  |  |  |  | 0.038 |
| Missing | 198 | (18.3) | 172 | (17.6) | 36 | (16.4) |  |
| >99.999 copies/mL | 134 | (12.4) | 102 | (10.5) | 14 | (6.4) |  |
| 10.000-99.999 copies/mL | 370 | (34.2) | 308 | (31.6) | 75 | (34.3) |  |
| ≤9.999 copies/mL | 379 | (35.1) | 394 | (40.4) | 94 | (42.9) |  |
| **cART duration^4^** |  |  |  |  |  |  | 0.931 |
| <9 months | 40 | (3.7) | 36 | (3.7) | 10 | (4.6) |  |
| 9-18 months | 86 | (8.0) | 77 | (7.9) | 20 | (9.1) |  |
| >18 months | 955 | (88.3) | 863 | (88.4) | 189 | (86.3) |  |
| **First-line cART^5^** |  |  |  |  |  |  | <0.001 |
| Yes | 226 | (20.9) | 116 | (11.9) | 35 | (16.0) |  |
| No | 855 | (79.1) | 860 | (88.1) | 184 | (84.0) |  |

Abbreviations: LLV, low-level viraemia; VF, virological failure; VL, viral load; NRTI, nucleoside reverse transcriptase inhibitor; NNRTI, non-nucleoside reverse transcriptase inhibitor; PI/r, boosted protease inhibitor; INSTI, integrase inhibitor; BLD, below the limit of detection; BLQ, below the level of quantification; cART, combination antiretroviral therapy;

^1^ Comparison between regimens.

^2^ Interruptions prior to 6 months stable cART of the respective cART regimen.

^3^ Diabetes Mellitus prior to 6 months stable cART of the respective cART regimen.

^4^ cART duration until 6 months stable cART of the respective cART regimen.

^5^ Whether the respective cART regimen is a first-line cART or not.
