# Supplementary material for: Factors Associated with Low-Level Viraemia and Virological Failure: Results from the Austrian HIV Cohort Study
Source: PLoS One. 2015 Nov 13;10(11):e0142923. doi: 10.1371/journal.pone.0142923 (PMC4643888; doi:10.1371/journal.pone.0142923)
Supplement: S2 Table — Exclusion of the samples in which the Abbott assay was used. (DOCX) [file pone.0142923.s002.docx]

**S2 Table: Univariable and multivariable logistic regression results: Association between different factors and low-level viraemia as well as virological failure compared to HIV RNA levels below the limit of quantification. Exclusion of the sample in which the Abbott assay was used.**

| Outcome | **LLV <200** | | | | **VF ≥200** | | | |
| --- | --- | --- | --- | --- | --- | --- | --- | --- |
| No. of patients included | N=1696 | | | | N=1576 | | | |
| No. of outcomes | N=199 | | | | N=79 | | | |
|  | **Univariable** | | **Multivariable** | | **Univariable** | | **Multivariable** | |
|  | **OR** | **(95% CI)** | **OR** | **(95% CI)** | **OR** | **(95% CI)** | **OR** | **(95% CI)** |
| **Age at viral load measurement** |  |  |  |  |  |  |  |  |
| <30 years | 1.18 | (0.69-2.02) | 1.03 | (0.58-1.82) | 3.50 | (1.35-9.04) | 2.65 | (0.99-7.09) |
| 30-50 years | 0.99 | (0.68-1.35) | 0.96 | (0.67-1.36) | 3.02 | (1.43-6.38) | 2.52 | (1.17-5.43) |
| >50 years | 1.00 | (Reference) | 1.00 | (Reference) | 1.00 | (Reference) | 1.00 | (Reference) |
| **HIV transmission category** |  |  |  |  |  |  |  |  |
| Male injecting drug user | 1.21 | (0.74-1.98) | 1.06 | (0.64-1.77) | 3.70 | (1.89-7.25) | 2.74 | (1.37-5.49) |
| Female injecting drug user | 0.65 | (0.25-1.68) | 0.55 | (0.21-1.46) | 2.80 | (1.01-7.77) | 2.25 | (0.79-6.44) |
| Male heterosexual | 0.79 | (0.53-1.19) | 0.78 | (0.52-1.19) | 0.90 | (0.41-1.94) | 0.73 | (0.32-1.66) |
| Female heterosexual | 0.66 | (0.43-1.00) | 0.67 | (0.43-1.04) | 2.06 | (1.12-3.78) | 1.15 | (0.57-2.29) |
| Other | 1.40 | (0.74-2.65) | 1.49 | (0.78-2.87) | 1.39 | (0.40-4.80) | 1.37 | (0.39-4.84) |
| Men who have sex with men | 1.00 | (Reference) | 1.00 | (Reference) | 1.00 | (Reference) | 1.00 | (Reference) |
| **Nationality** |  |  |  |  |  |  |  |  |
| High prevalence country | 1.23 | (0.77-1.99) |  |  | 2.33 | (1.29-4.19) | 2.31 | (1.14-4.67) |
| Low prevalence country | 1.00 | (Reference) |  |  | 1.00 | (Reference) | 1.00 | (Reference) |
| **CD4 count before cART** |  |  |  |  |  |  |  |  |
| Missing | 1.38 | (0.84-2.26) |  |  | 0.86 | (0.41-1.77) |  |  |
| <50 cells/µL | 1.09 | (0.61-1.93) |  |  | 0.48 | (0.18-1.29) |  |  |
| 50-199 cells/µL | 1.37 | (0.86-2.18) |  |  | 0.95 | (0.49-1.84) |  |  |
| 200-349 cells/µL | 1.27 | (0.83-1.94) |  |  | 0.82 | (0.45-1.50) |  |  |
| ≥350 cells/µL | 1.00 | (Reference) |  |  | 1.00 | (Reference) |  |  |
| **Ever cART interruptions^1^** |  |  |  |  |  |  |  |  |
| ≥1 | 1.56 | (1.12-2.16) | 1.66 | (1.17-2.35) | 3.26 | (2.07-5.16) | 2.91 | (1.81-4.68) |
| None | 1.00 | (Reference) | 1.00 | (Reference) | 1.00 | (Reference) | 1.00 | (Reference) |
| **Ever diabetes^2^** |  |  |  |  |  |  |  |  |
| Yes | 1.51 | (0.80-2.86) |  |  | 0.61 | (0.15-2.55) |  |  |
| No | 1.00 | (Reference) |  |  | 1.00 | (Reference) |  |  |
| **cART regimen** |  |  |  |  |  |  |  |  |
| 2 NRTI + PI/r | 1.66 | (1.24-2.24) | 1.60 | (1.18-2.18) | 2.88 | (1.80-4.62) | 2.27 | (1.39-3.71) |
| 2 NRTI + NNRTI/INSTI | 1.00 | (Reference) | 1.00 | (Reference) | 1.00 | (Reference) | 1.00 | (Reference) |
| **Viral load before cART** |  |  |  |  |  |  |  |  |
| Missing | 2.59 | (1.21-5.53) | 2.37 | (1.09-5.12) | 1.15 | (0.47-2.82) |  |  |
| >99.999 copies/mL | 4.06 | (2.01-8.20) | 3.95 | (1.94-8.04) | 1.20 | (0.53-2.70) |  |  |
| 10.000-99.999 copies/mL | 2.41 | (1.17-4.96) | 2.38 | (1.15-4.94) | 1.50 | (0.68-3.31) |  |  |
| ≤9.999 copies/mL | 1.00 | (Reference) | 1.00 | (Reference) | 1.00 | (Reference) |  |  |
| **cART duration^3^** |  |  |  |  |  |  |  |  |
| <9 months | 2.00 | (1.09-3.68) | 2.54 | (1.32-4.90) | 0.70 | (0.17-2.94) |  |  |
| 9-18 months | 0.90 | (0.52-1.55) | 0.97 | (0.55-1.71) | 1.11 | (0.52-2.35) |  |  |
| >18 months | 1.00 | (Reference) | 1.00 | (Reference) | 1.00 | (Reference) |  |  |
| **First-line cART^4^** |  |  |  |  |  |  |  |  |
| Yes | 0.79 | (0.53-1.18) |  |  | 0.48 | (0.23-1.01) |  |  |
| No | 1.00 | (Reference) |  |  | 1.00 | (Reference) |  |  |

Abbreviations: LLV, low-level viraemia; VF, virological failure; VL, viral load; NRTI, nucleoside reverse transcriptase inhibitor; NNRTI, non-nucleoside reverse transcriptase inhibitor; PI/r, boosted protease inhibitor; INSTI, integrase inhibitor; BLD, below the limit of detection; BLQ, below the level of quantification; cART, combination antiretroviral therapy;

^1^ Interruptions prior to 6 months stable cART of the respective cART regimen.

^2^ Diabetes Mellitus prior to 6 months stable cART of the respective cART regimen.

^3^ cART duration until 6 months stable cART of the respective cART regimen.

^4^ Whether the respective cART regimen is a first-line cART or not.
